# Capital-income breeding in wild boar: a comparison between two sexes

**DOI:** 10.1038/s41598-021-84035-w

**Published:** 2021-02-25

**Authors:** Rudy Brogi, Roberta Chirichella, Francesca Brivio, Enrico Merli, Elisa Bottero, Marco Apollonio

**Affiliations:** 1 Department of Veterinary Medicine, University of Sassari, via Vienna 2, 07100 Sassari, Italy; 2 Agriculture and Wildlife Service, Emilia-Romagna Region, via Garibaldi 50, 29122 Piacenza, Italy

**Keywords:** Ecology, Evolution, Zoology

## Abstract

Organisms differ in the strategy adopted to fuel reproduction by using resources either previously acquired and stored in body reserves (capital breeding) or, conversely, acquired during their reproductive activity (income breeding). The choice of one or the other strategy is related to several internal and external factors which are counteractive in wild boar. Based on a large dataset of culled wild boar, we investigated individual body weight variability throughout the period of 1st September–31st January, which included the main part of the mating season, among different sex and age classes to determine their position along the capital-income breeding continuum. Though food resources were abundant during the rut, adult males lost body weight suggesting they adopted a predominantly capital breeding strategy, likely owing to the high intra-sexual competition entailed by the peculiar mating system of the species. On the contrary, subadult males seemed to behave as income breeders, likely enhancing the reproductive flexibility of wild boar populations. During the rut, females stored reserves, thus suggesting that they substantially relied on them to cover future reproductive costs.

## Introduction

The life history of an animal is comprised of sets of trade-offs among growth, survival, and reproduction that organisms face during their life^1^. A major aspect of life history diversity among animal species is that the resources allocated to reproduction are obtained either from stored reserves within the body or the current intake, resulting in the division between capital and income breeders (e.g.,^2,3^). This concept is of utmost importance in theoretical evolutionary ecology as it influences both the body condition-reproductive success relationship and the time lag of organisms-environmental resources linkage^3^, but it can also be profitably applied to conservation and management as it affects a species’ sensitivity to environmental changes^3,4^. Furthermore, given the wide exploitation of ungulates in hunting and their growing involvement in wildlife-human conflicts, their life histories are raising a strong interest among researchers.

Stephens and colleagues^3^ reported that the degree of capital and income breeding of organisms is related to a variety of ecological, morphological, and physiological factors. In particular, high food availability during the breeding season typically promotes income breeding strategies^2^, while temporal mismatches between resource supply and reproductive demand promote capital breeding^5^. Larger body size can facilitate capital breeding on account of a lower relative cost for reserve transportation and a higher metabolism efficiency^3^. The mating system and, specifically, the degree of polygyny may act as a further push-factor in positioning male ungulates along the capital-income continuum^6^. Indeed, higher levels of intra-sexual male competition for mating opportunities are likely to enhance the reproductive demands of polygynous males and, concomitantly, their tendency to adopt feeding reduction^7,8^ and suppression^9,10^ during the rut, inevitably forcing them to rely on a stored capital of reserves. Moreover, in order to maximize their lifetime reproductive success, individuals can occupy different positions along the capital-income breeding continuum throughout their life cycles^5^. Indeed, adult male ungulates typically show high body weight loss during the rut (i.e., high reliance on stored reserves) compared to younger males which, conversely, give priority to growth. As a consequence, young males show a limited or null body weight loss (for a review, see Mysterud et al.^11^), although they can still be fully or partially involved in reproduction^12,13^. When evaluating life history strategies, it is therefore essential to first characterise sex and age classes, as groups of individuals at different stages of the growth-reproduction trade-off are likely to adopt different strategies for the acquisition of resources to invest in reproduction.

One of the major constraints for studies on capital-income breeding lies in the difficulty to objectively circumscribe the time period over which the reproductive costs should be measured^3^. As female investment into reproduction usually includes a variety of activities linked to a single reproductive event (for mammals: mating, foetuses growth, giving birth, and lactation), studies on females are particularly concerned with the difficulties in circumscribing the period of such reproductive costs. Conversely, since most male ungulates have no further reproductive cost after conception^11^, their reproductive effort is entirely included in the rutting season. Nevertheless, finding an objective way to circumscribe this period on a local level entails several practical complications. Previous studies on male ungulate life histories arbitrarily delimited the rut^14^, obtained it from other studies^15^, or roughly derived it from field behavioural observations^16^.

As a rare example of highly polygynous species^17^, exhibiting similar early-life growth rates in both sexes (e.g.,^18^) and a short generation time compared to other ungulates (e.g.,^19,20^), wild boar (*Sus scrofa*) is a particularly interesting species for studying life history strategies. The position of wild boar along the capital-income breeding continuum has previously only been determined for females and was found to be different according to the area and the study^19,21,22^, with litter size being the only measure of reproductive effort considered. Conversely, males’ reliance on the stored capital or the available resource income for reproduction has never been investigated, though this sex presents a unique combination of contrasting factors pushing simultaneously towards the two opposite strategies. On the one hand, the mating period occurs when food resources are relatively abundant. This should prevent the need of previously stored energy and facilitate income breeding. Oak (*Quercus* spp.), chestnut (*Castanea sativa*), and beech (*Fagus sylvatica*) seed production, which accounts for most of European wild boar’s diet (at least in natural and semi-natural situations in which agricultural crops are scarce and supplementary feeding is not provided^23,24^), is typically concentrated in late autumn, when mating usually occurs^25^. On the other hand, wild boar morphology and reproductive biology should push males to adopt capital breeding, by reducing costs associated with this strategy and accounting for feeding suppression, respectively. As a matter of fact, large size and a thermally efficient body shape (sensu Allen^26^) should enhance wild boar metabolism efficiency, thus reducing costs of capital storing, transportation, and maintenance. The relatively high degree of polygyny of this species^17^ entails high competition among males for mating opportunities. This may be expected to increase both the need and the potential reproductive value of relying on stored reserves and thus promote capital breeding^6^. In addition, the gregarious habits of females^27^ and the high litter size^28^ make male reproductive effort even more beneficial in terms of potential number of descendants, thus exacerbating intra-male competition.

Age can also be expected to heavily determine individual strategies to fuel reproduction, as younger wild boar still need to allocate part of the resources to growth. Consequently, they have lower body reserves to invest^29^. As mentioned above, it becomes essential to discuss individuals’ reproductive reliance on stored reserves in the context of their growth stages, typically represented by age classes. Nevertheless, the available growth curves on wild boar are provided by studies limited by the use of either a small sample size^30,31^, or descriptive statistics alone^18^, or both^32,33^.

When relatively high, hunting pressure can also play a role in shaping wild boar reproductive strategies, as an unbalanced removal of adult individuals can influence the first reproduction of both subadult males^34^ and females^19^. If the harvest is adult male-biased (not the case of our study area^35^), hunting can also cause a shortage of adult males and, therefore, lower the levels of sexual competition^36^, thus potentially reducing the reproductive effort and ultimately the need of capital breeding. Nevertheless, an opposite effect (i.e., increased male reproductive costs) was described by Mysterud et al.^14^ in female-skewed moose (*Alces alces*) populations, likely because males had to enhance their displacements in order to take advantage of the higher number of available female groups.

Based on a large dataset of culled wild boar, we first modelled male and female body growth curves and identified age classes in order to properly compare breeding strategies among homogeneous groups of individuals. To independently determine the period over which male reproductive effort is sustained, we assessed female oestrus distribution throughout the year and used it as a proxy of the rutting season. We then compared body weight variability throughout autumn–winter in different sex and age classes in order to evaluate potential changes in male weight with respect to other classes owing to their reproductive effort. In so doing, we aimed to ascertain their position along the capital-income breeding continuum.

## Results

### Sex and age class identification

**Figure 1:**
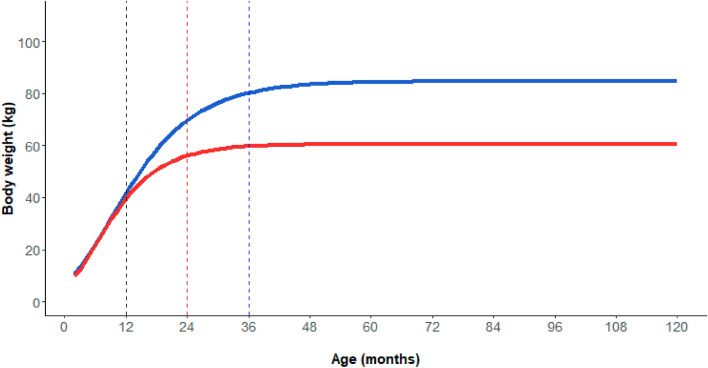
Body weight variation of males (blue line) and females (red line) at growing ages. Values were predicted by the Gompertz growth models separately for males and females (see the text for more details). Vertical dashed lines represent the limits between piglets-subadults (both sexes, black line), subadult-adult females (red line), subadult-adult males (blue line).

Gompertz growth models’ estimated parameters, summarised in Supplementary Table S1, were all statistically significant. Sexual size dimorphism appeared around 1 year of age. Males had to reach 3 years of age to exceed 90% of their asymptotical weight (85 kg), while the age for females was 2 years (female asymptotical weight = 61 kg, Fig. 1). On this basis, the following sex and age classes were identified: male and female piglets (individuals younger than 1 year), subadult males (males older than 1 year but younger than 3 years), subadult females (females older than 1 year but younger than 2 years), adult males (males older than 3 years), and adult females (females older than 2 years). Sample distribution among sex and age classes is reported in Supplementary Table S2.

### Rutting season identification

**Figure 2:**
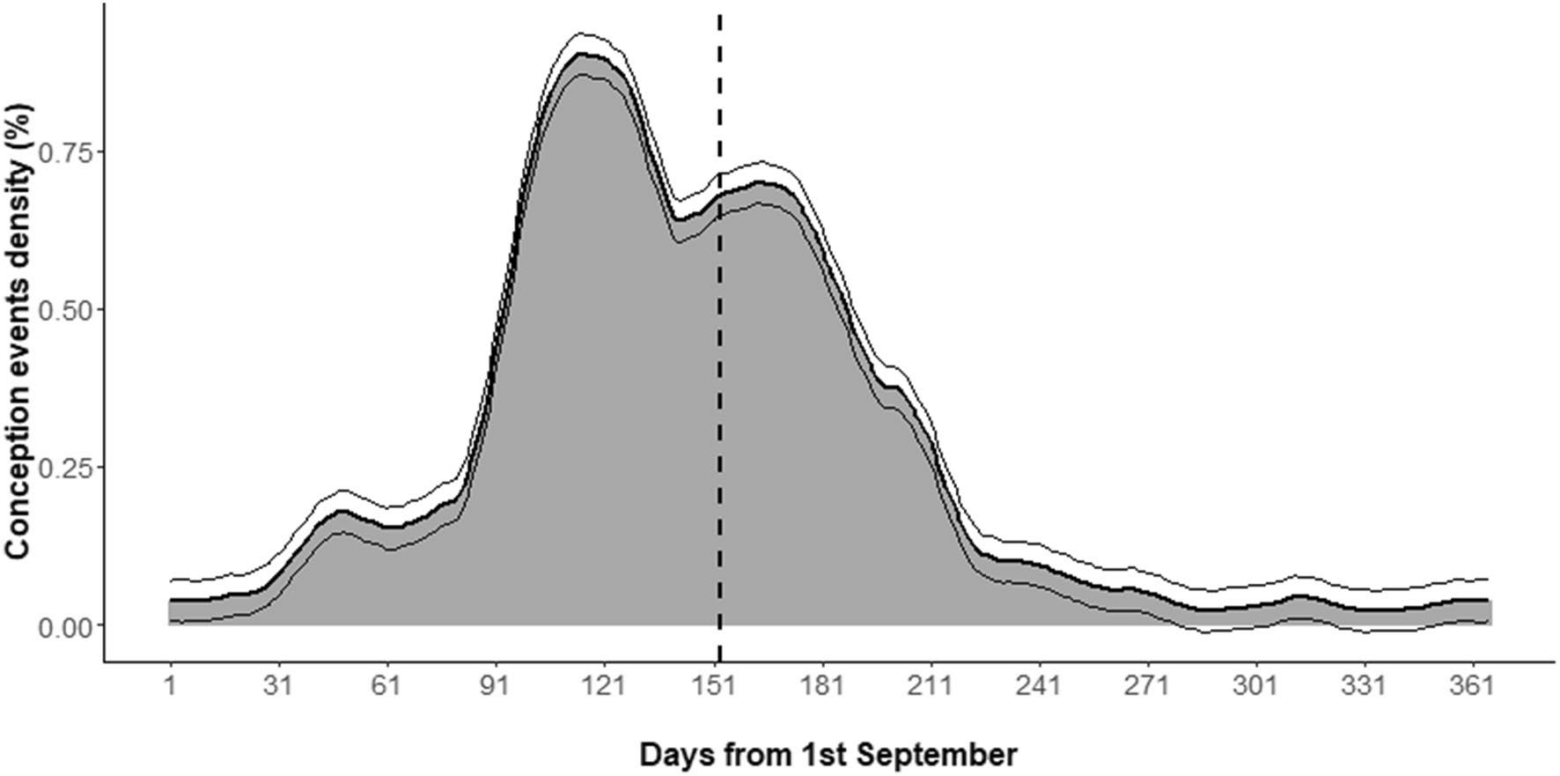
Conception event smoothed distribution throughout the year assessed from individual age of piglets and subadult individuals, culling date, and gestation period (see the text for more details). Upper and lower thin lines represent the distribution of mean + SE and mean − SE, respectively. Date is expressed as days from 1st September and equivalent to the sampling day. The dashed line represents the end of the sampling period (153 days, from 1st September to 31st January).

The intra-annual distribution of conception dates started in October, peaked in January and lasted until April, with most events concentrated in the period December-March (Fig. 2). The portion of conception events occurring during the sampling period (153 days starting from 1st September) was 59.68 ± 5.00% (mean ± SE) of the total.

### Seasonal variability of individual body weight in different classes

**Figure 3:**
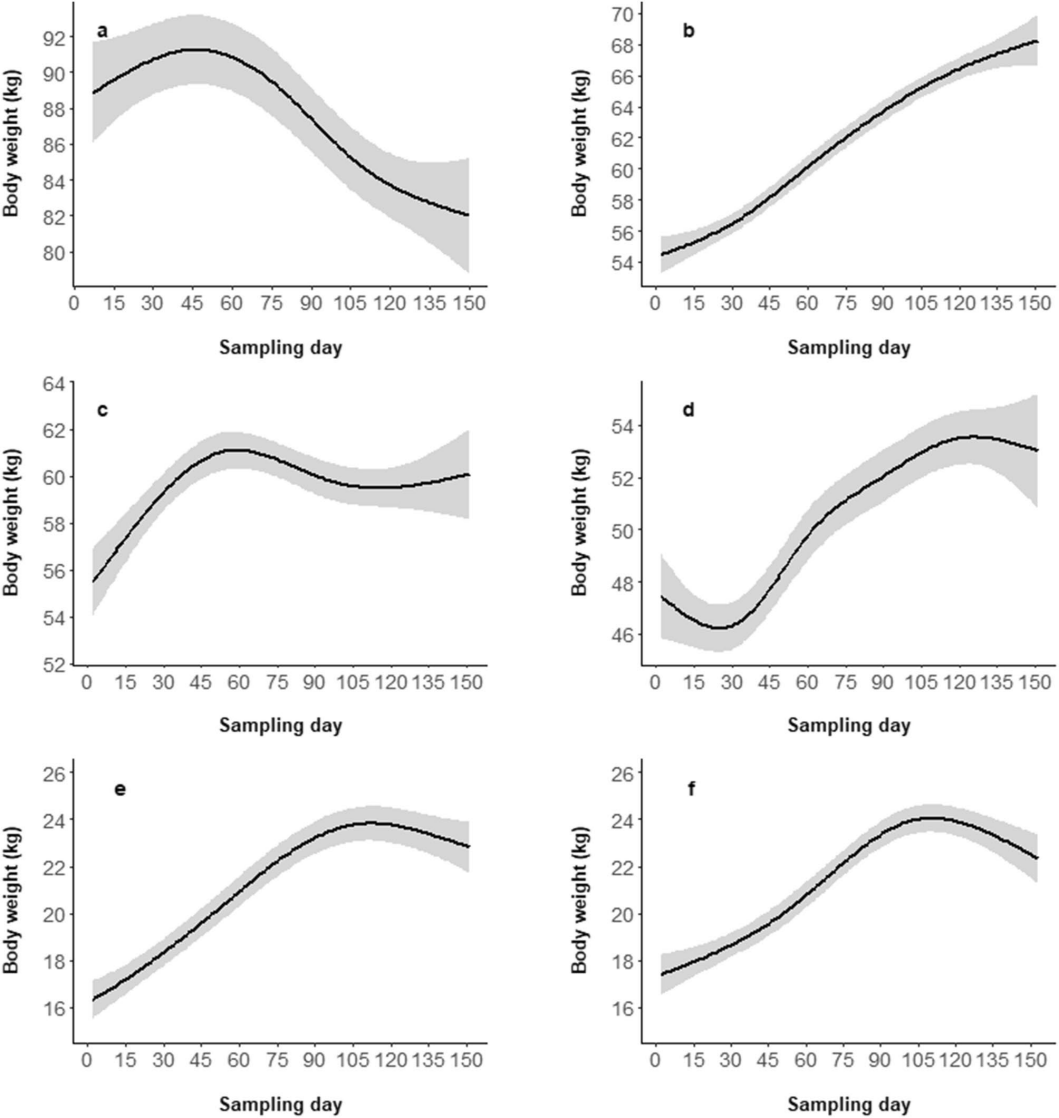
Body weight variation of adult males (**a**), adult females (**b**), subadult males (**c**), subadult females (**d**), male piglets (**e**), and female piglets (**f**) throughout the sampling period. The first sampling day corresponds to 1st September. Values were predicted by the best models separately for each class. Grey-shaded areas represent the estimated standard errors. The predictions are given according to the mean of all other covariates in the models.

All selected best models (identified following the minimum Akaike’s Information Criterion, AIC, see Methods for more details) significantly explained body weight variability (p-values of all included predictor are reported in Supplementary Tab. S3). Adult males' best model included sampling day, individual age, previous winter rain precipitation, and spring temperature as predictor variables (R^2^_adj_ = 0.100). Throughout the sampling period, adult males showed a non-linear pattern of body weight variability, with a slight increase during the first part of the sampling period (lasting approximately 50 days) and a subsequent steady loss. Predicted weights ranged from a maximum of about 91 kg (around the 50th day of the sampling period) to 82 kg (at the end of the sampling period, Fig. 3a), thus showing a weight loss of 9.89%. As they grew older, adult males showed only a slight, constant weight gain. Adult male weights increased with increasing spring average temperature, until reaching a maximum peak with an average temperature of 8.0 °C, then slightly decreased above this optimal value, and finally stabilised above 9.5 °C. A slightly positive effect of previous winter rain precipitation was detected (see Supplementary Fig. S1).

The best model explaining adult female body weight variability included sampling day, individual age, and previous year chestnut productivity as predictor variables (R^2^_adj_ = 0.214). Adult females gained body weight with a steady pattern throughout the sampling period, starting with an average weight of 55 kg and reaching up to 68 kg at the end of the period (Fig. 3b), with a total gain of 23.64% of the initial weight. In accordance with the results of the Gompertz body growth model, adult females showed substantially stable weights at growing ages. Though statistically significant, previous year chestnut productivity had a positive but biologically negligible effect on adult female body weight (Supplementary Fig. S2).

The best model predicting subadult male body weight variability included the following set of predictor variables: sampling day, individual age, previous year chestnut productivity, previous winter, spring, and summer average temperatures (R^2^_adj_ = 0.238). Their body weight showed only small variations throughout the sampling period (predicted values: 55–61 kg), with an initial slight increase lasting about 50 days, followed by a horizontal pattern lasting for the rest of the season (Fig. 3c). Individual age had a clear, positive effect on the predicted body weight, while previous year chestnut productivity accounted for slightly higher body weight. Finally, the average temperature of the summer and spring months preceding the hunting season negatively affected subadult male body weight, while that of the previous winter months did not show any relevant effect (Supplementary Fig. S3).

Subadult female body weight variability was explained by the best model including sampling day, individual age, previous summer average temperature, previous winter rain precipitation, and current autumn rain precipitation as predictor variables (R^2^_adj_ = 0.233). Females of this age class showed a steady increase of their body weight throughout the sampling period, a result which is similar to that of adult females, though with wider confidence intervals (Fig. 3d). Moreover, the relation with age was positive. As with subadult males, the best model predicted a substantial negative relation between body weight and previous summer average temperature. Subadult females reached their maximum body weight with mean values of rain precipitation during the previous winter (around 4 mm/day), while higher values of current autumn rain (above 5.0 mm/day) accounted for heavier body weights (Supplementary Fig. S4).

The best model explaining the variability of male piglet body weight included the predictors: sampling day, individual age, current year global productivity index, mean rain precipitation of previous summer, and average temperature of previous spring (R^2^_adj_ = 0.370). In this class, body weight increased with a steady pattern throughout the sampling period until the 110th sampling day and slightly decreased during last 40 days of hunting (Fig. 3e). Individual age had a positive effect on the response variable, with older male piglets being constantly heavier than younger ones. The relation between male piglet body weight and current year global productivity index was linear and positive, whereas other predictor variables had a significant but biologically negligible effect (Supplementary Fig. S5).

As for female piglets, the best model included sampling day, individual age, and previous year Turkey oak (*Quercus cerris*) productivity as predictor variables (R^2^_adj_ = 0.331). Their predicted body weight increased throughout the sampling period, with a pattern essentially identical to that of male piglets (Fig. 3f). Likewise, a positive effect of individual age was assessed. Finally, female piglet body weight was higher when previous year Turkey oak productivity was around 0.4 Mg/ha (Supplementary Fig. S6).

## Discussion

We investigated wild boar capital-income breeding strategies by using a large dataset of culled individuals. We objectively characterised age classes and quantitatively assessed the timing of the rut with a large sample of conception dates and a comprehensive account of uncertainty. Our results suggest that adult males relied on a stored capital of reserves to cope with their reproductive requirements, although weight gains of other classes confirmed the expectation that food resources were particularly abundant during the rut.

Our sex and age classification on the basis of growth stages is consistent with that used in previous studies with regards to piglets of both sexes and females in general^19,25,36^. On the contrary, the subdivision between subadult and adult males was placed at 3 years, unlike other studies (2 years in^33,34,36^). As males were clearly still growing between 2 and 3 years of age, they could not afford a full investment in reproduction, despite being already sexually mature^37^, which is the typical condition of subadults. In this respect, we would argue that our classification better generalised male growth stages. This enabled us to properly compare body weight variation patterns and breeding strategies among homogenous groups of individuals.

Only adult males showed an absolute weight loss during the sampling period (1st September–31st January), whereas all other classes gained body weight, though with different extents and patterns (Fig. 3). Food resources were particularly abundant during that time of the year, as confirmed by weight gains of other classes as well as by data referring to wild boar spatial behaviour within the same area^38^. Since hunting disturbance is known to have a minimal impact on wild boar behaviour^39,40^ and the rich-food habitats (forest) are also the safest refuges from hunting risk in our study area^35^, we can exclude the possibility that hunting affected the weight loss observed in adult males. Reproductive efforts were more likely to be the main cause of this negative trend, as supported by the temporal match between the start of adult male body weight decrease (around the 50th sampling day) and the start of the conception event distribution. We may directly estimate a relative loss of about 9.89% of the pre-reproductive adult male body weight (50th sampling day), though the total weight loss related to reproduction was likely much higher. Indeed, our sampling period was constrained by hunting season limits and covered only a part of the rutting season, including 59.68 ± 5.00% of all conception events (Fig. 2). If the relation between body weight loss and conception event distribution had remained the same as it was observed during the sampling period, we can estimate that adult males would have lost 16.57 ± 1.39% of their pre-reproductive body weight by the end of the rut. Adult male wild boar relative weight loss estimated by our analysis can be compared with that of male Alpine chamois (*Rupicapra rupicapra rupicapra*, 17–19% in Mason et al.^16^ and 16.0% in Apollonio et al.^6^) and male red deer (*Cervus elaphus*, 19.5% in Apollonio et al.^6^), which are usually considered capital breeders^6,14^. Accordingly, our results position adult male wild boar towards the capital end of the capital-income breeding continuum.

Both the reproduction effort itself and feeding reduction or suppression during the rutting season possibly accounted for the reproduction-induced weight loss of adult males. Though information on male wild boar reproductive behaviour is still lacking, during the rut they are thought to roam widely in search for groups of receptive females, actively competing to monopolise and finally mate with them^41,42^. This behavioural pattern is likely to enhance the energetic expenditure of males during the rut. Even though hunting pressure may partially weaken the direct competition to monopolise female groups by unbalancing the population structure toward females^36^, a female-skewed population has been shown to increase male reproductive cost in other species (e.g., in moose^14^). This is likely due to a higher energy expenditure in spatial movements, as each male would have the opportunity to mate with several scattered female groups. Nevertheless, in such a food-rich season, the massive weight loss observed can hardly be explained by energy expenditure alone. However, the almost total feeding suppression which characterises a number of male polygynous ungulates (see Miquelle^9^ for moose; Apollonio and Di Vittorio^10^ for fallow deer, *Dama dama*) would be unaffordable for wild boar, given the long-lasting rut. Indeed, it was never detected in studies involving the analysis of wild boar stomach content^23,43^. We can therefore presume that adult male wild boar may adopt milder forms of feeding reduction during the rut, similarly to male Alpine ibex (*Capra ibex*)^7^ and Alpine chamois^8^. This explanation is supported by the decrease of the insulin-like growth factor 1 concentrations (IGF-I, whose secretion is linked with energy supply) observed in males during autumn and winter by Treyer et al.^43^. This may have also contributed to weaken the effect of food abundance during the rut in determining the adoption of an income breeding strategy, by preventing individuals to fully exploit it.

Similarly to adult males, subadult males increased their body weight during the first part of the sampling period but then showed substantially stable values, with an almost flat slope (Fig. 3c). As they are still growing, subadult males may not have considerable stored reserves available for reproduction. The temporary 2–3 month growth break observed may indicate that subadult males took part in reproduction (as previously suggested by Šprem et al.^34^), though investing only resources from the current intake and thus behaving as income breeders. Since income breeding can only support a small reproductive investment and a direct competition with adults would be totally ineffective for them^44^, we can argue that subadult males relied on alternative mating tactics to achieve at least some paternities^12,13^. Wild boar social organisation may have also contributed to the missed weight gain observed in this class. Indeed, during the rut adult males display agonistic behaviours against subadult males joining females groups^27^, potentially moving them away from food-rich areas, which are typically occupied by females. Thus, we can argue that subadult males’ reproductive contribution is inversely dependent on the availability of adult males in the population. This may therefore potentially reduce the negative effect of a male-biased culling on the reproductive outcomes.

Both adult and subadult females gained body weight almost steadily during the whole sampling period (Fig. 3b,d). However, this result did not allow us to directly determine their position along the capital-income breeding continuum. Indeed, female reproductive investment can be considered negligible during the mating season, then becoming substantial during the subsequent phases of foetuses formation, birth, and weaning, which essentially occupy the rest of the year. While subadult females were still growing and therefore may have allocated the resources acquired during autumn–winter to body growth, adult females have already completed their body development and reasonably invested the resources stored during this period in the subsequent reproduction phases. This suggests that adult females substantially relied on reserves stored in autumn–winter to cover future reproductive costs and, thus, adopted a capital breeding strategy.

We used a long-lasting dataset sampled during 14 consecutive hunting seasons but limited to 5 months per year. This prevented us from properly evaluating females’ reproductive reliance on stored reserves and observing the last portion of the rutting season. However, we managed to predict the total reproductive cost carried by adult males by means of a quantitative and independent assessment of rut timing. Our large sample size provided a robust insight into wild boar life history at a population level, which would have been unfeasible with longitudinal studies as they are typically limited to few monitored individuals (e.g.,^12,45^ ). Nevertheless, further well-designed longitudinal studies may be extremely useful to evaluate the heterogeneity of wild boar life history on an individual level.

In conclusion, we demonstrated that adult male wild boar adopted a predominantly capital breeding strategy, while subadult males likely behaved as income breeders and enhanced the reproductive flexibility of the populations. Though we were not able to directly assess females’ strategy, we detected a strong resource storage during the mast period, which is likely to be invested in the subsequent reproduction effort. Being capital breeders generally less sensitive to environmental variability^3,4^, we can argue that wild boar reproductive outcomes will be highly resilient to ecological perturbations.

## Materials and methods

### Study area

Our study was conducted in the Alpe di Catenaia mountainous area (Northern Apennines, Italy, 43° 48′ N, 11° 49′ E, Supplementary Fig. S7) which covers a total surface of 13,400 ha and includes a protected area (Oasi Alpe di Catenaia) of 2,700 ha. Altitude ranges from 330 to 1,414 m above the sea level. The temperate-continental climate shows marked seasonal variations, with hot and dry summers (mean temperature of 18.7 °C and daily precipitation of 1.73 mm) and cold and rainy winters (mean temperature of 1.2 °C and daily precipitation of 3.55 mm). Snowfalls occur only occasionally between October and April. The area is mainly covered with mixed deciduous woods (67% of the total surface), with Turkey oak, beech, and chestnut as the most abundant tree species, while conifer woods (7%), agricultural crops (16%), and mixed open-shrubs areas (10%) cover the rest of the surface. Wild boar unselective drive hunts (i.e. targeting all social classes) involved 25–50 hunters and were performed in the surroundings of the protected area three times a week from September–October to January (on average of 58.3 hunting days per year). Hunting pressure was high and relatively constant over the years, with an average of 6.4 wild boar/km^2^ harvested every year^35^.

### Data collection

We collected data on 8,763 wild boar of all age and sex classes culled within our study area from 1st September to 31st January in the period 2002–2016, for a total of 14 consecutive hunting seasons. Undressed body weight and culling date were recorded for each wild boar. Since female reproductive traits were not fully available for measurements, we could not subtract foetus weight from pregnant female body weight, thus potentially overestimating their body condition. Nevertheless, foetus weight (calculated on a subsample of 415 pregnant females with measurable reproductive traits) accounted for a negligible portion of mother total body weight (on average 0.51 ± 0.95%, mean ± SD). On the basis of their tooth eruption and abrasion^46^, all wild boar were assigned to one of the following age intervals: < 3 months, 3–4 months, 5–6 months, 7–9 months, 10–12 months, 13–14 months, 15–16 months, 17–18 months, 19–20 months, 20–22 months, 22–24 months, 24–36 months, 3–4 years, 5–7 years, 8–10 years or > 10 years. Given the intrinsic characteristics of the tooth-based aging method, we are aware that precision decreased as age increased. Notwithstanding, this was the only feasible approach to age a large number of culled individuals.

Yearly seed productivity of beech, chestnut, and Turkey oak was acquired from an online database reporting local data collected in our study area^47^. Weather data were recorded daily in a weather station located inside our study area (43° 42′ N, 11° 55′ E) and kindly provided by the Regional Hydrological Service of Tuscany.

### Ethical declarations

Data collection did not involve any alive animal. All wild boar included in analysis were culled according to Italian national and regional hunting laws.

### Data analysis

#### Sex and age class identification

As we aimed to assess patterns of body growth of both sexes during different age stages, we distinguished culled individuals into males and females, thus creating 2 sub-datasets out of our original dataset (males, n = 4398, and females, n = 4365). We then assigned individual ages as the median of the age interval identified by means of tooth analysis. For each sub-dataset, body growth was then described by fitting weight to age with the Gompertz growth equation^45,48,49^ through a 3-parameter nonlinear model:

$$W=a*{e}^{-b{e}^{-cx}}$$

in which *W* is body weight at age *x*, *a* is the asymptotic body weight, *e* is the exponential constant, *b* is the displacement on the x-axis, and *c* is growth rate. We estimated *a*, *b* and *c* by means of the *SSgompertz* function of the *stats* package in R 3.2.2^50^. Finally, we used the growth curves obtained to identify 2 breakpoints: (i) age of sexual size dimorphism appearing and (ii) age of body weight exceeding 90% of its asymptotic value (sex-specific), rounding them on a yearly basis to correctly distinguish cohorts. Depending on their individual age, male and female wild boar were separately grouped into 3 age classes: piglets (below first breakpoint), subadults (above the first and below the second breakpoint) and adults (above the second breakpoint).

#### Rutting season identification

In order to identify the rutting season for the studied population, we estimated the temporal distribution of conception events. Individual conception dates were estimated from the age of culled piglet and subadult wild boar, culling date and gestation period, following the formula:

$$CoD=CuD-IA-GP$$

with *CoD* being the conception date, *CuD* the culling date, *IA* the individual age expressed in days of the culled wild boar, and *GP* an average gestation period of 118 days (obtained as the mean between a gestation period of 115 days reported by Henry^51^ and of 121 days reported by Vericad^52^). *IA* was estimated as the median of the age interval identified. Only wild boar aged 2 years or younger were included in analyses, as their age interval width was ≤ 3 months, for a total of 6604 individuals. In order to take into account both sources of uncertainty (gestation period and ageing process), we smoothened the number of conception events occurring per date by means of the *loess* function of the *stats* package in R. We used a 41-day span width, i.e., the average standard error of conception date attribution, which was calculated as 1/1.96 of the sum of the mean age interval width (74 days) and the 6-day difference between two conception periods. Finally, we quantified the portion of conception events which occurred during our sampling period.

#### Seasonal variability of individual body weight in different classes

In order to evaluate the variability of individual body weight throughout the sampling period and its relation with reproduction efforts, we divided our dataset into 6 sub-datasets corresponding to sex and age classes previously identified by means of body growth models (adult males, n = 752, adult females, n = 1376, subadult males, n = 1629, subadult females, n = 1318, male piglets, n = 2017, and female piglets, n = 1671). Individual body weight was modelled by means of Generalised Additive Models (GAMs) with a Gaussian distribution, which were implemented by means of the *mgcv* package in R, separately for each sub-dataset. Sampling day was standardised as the number of days from 1st September and used as predictor to observe the variability of individual body weight throughout the sampling period. In order to enhance the models’ robustness, we also included individual age, previous and current year forest productivities, and weather variables as predictors. Individual age, expressed in months, was calculated as the median of the age interval identified by means of the tooth analysis and used to take into account the residual age-related source of variation in individual body weight. Current and previous year productivity of Turkey oak, beech, and chestnut, expressed as Mg/ha, were measured on a yearly basis and included in the models to consider inter-annual variability of food resource availability and its potential effect on individual body weight. Moreover, we included a global forest productivity index, which was calculated as the sum of the relative productivity of all three species, which were in turn obtained as the ratio of the productivity of a certain tree species in a given year over the mean productivity of the same species during the entire study period^38^. Finally, to account for the potential indirect effect of weather on individual body weight of wild boar, we included the seasonal average of temperature and rain precipitation in the models. Since all individuals were culled during the hunting season of year x, seasonal temperature and seasonal rain precipitation were calculated on a yearly basis with the following rule: weather variables were averaged from December of year x-1 to February of year x in winter, from March to May of year x in spring, from June to August of year x in summer, and from September to November of year x in autumn. Values of the 8 weather variables (average temperature and average daily rain precipitation for each of the four seasons) were then assigned to each individual according to the hunting season of culling. For each sub-dataset discretely, predictors were screened for collinearity (Pearson correlation matrix, rp) and multicollinearity (Variance Inflation Factor), with thresholds set to r_p_ =  ± 0.7 and VIF = 3, respectively^53^. Among the different sub-datasets, the most recurring groups of variables affected by collinearity included forest productivities of the same year, especially the chestnut-Turkey oak and beech-global index productivity pairs, and mean temperature and daily precipitation of the same season, particularly spring and autumn. To select the best candidate predictors among the collinear variables, we screened them by means of a machine learning method, the random forest calculation (*random.Forest* package), which ranked all predictor variables on the basis of their potential to explain body weight variability^54^. We dropped the worst predictor variable of each collinearity condition until no variable affected by multicollinearity remained.

The final step of analysis consisted of a model selection process for each sub-dataset. We built a full GAM which included all the predictor variables selected in the previous step, with the effect of all variables modelled as a natural cubic spline function. Subsequently, we used the dredge function of the *MuMln* package to run a set of models with all possible combinations of the full model predictor variables. The best models were then identified following the minimum AIC and the most parsimonious (in terms of number of predictor variables included) were selected in case of pairs and groups of models with ΔAIC < 2^55^. We performed a validation of the models selected by visually inspecting their residuals to check for homoscedasticity, normality of errors, and independence^53^.

## Supplementary Information

Supplementary Information.

## Data Availability

The dataset analysed during the current study is available from the corresponding author on reasonable request.
