# Supplementary material for: Capital-income breeding in wild boar: a comparison between two sexes
Source: Sci Rep. 2021 Feb 25;11:4579. doi: 10.1038/s41598-021-84035-w (PMC7907357; doi:10.1038/s41598-021-84035-w)
Supplement: Supplementary file 1 — Supplementary Information. [file 41598_2021_84035_MOESM1_ESM.pdf]

Capital-income breeding in wild boar: a comparison between two sexes

R. Brogi, R. Chirichella, F. Brivio, E. Merli, E. Bottero, and M. Apollonio

SUPPLEMENTARY INFORMATION

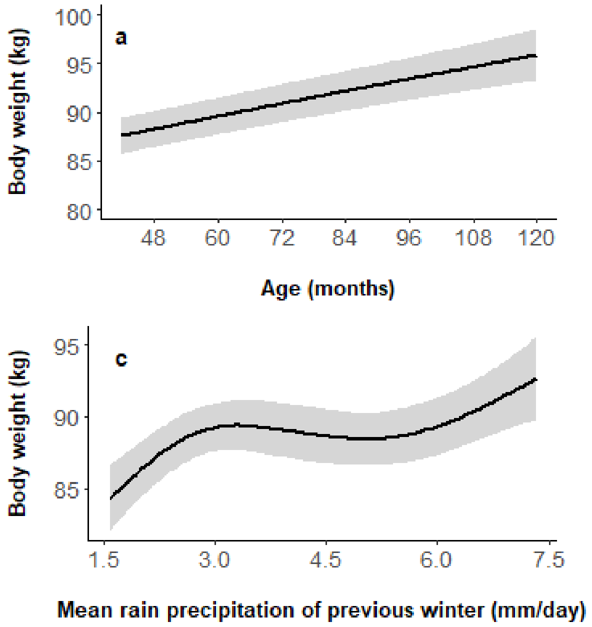

Fig. S1. Effect of other predictor variables (a: individual age, b: average temperature of previous spring, c: mean rain precipitation of previous winter) included in the best generalised additive mixed model explaining adult male body weight variation throughout the hunting season (see the text for more details). Grey-shaded areas represent the estimated standard errors. The predictions are given according to the mean of all other covariates in the model.

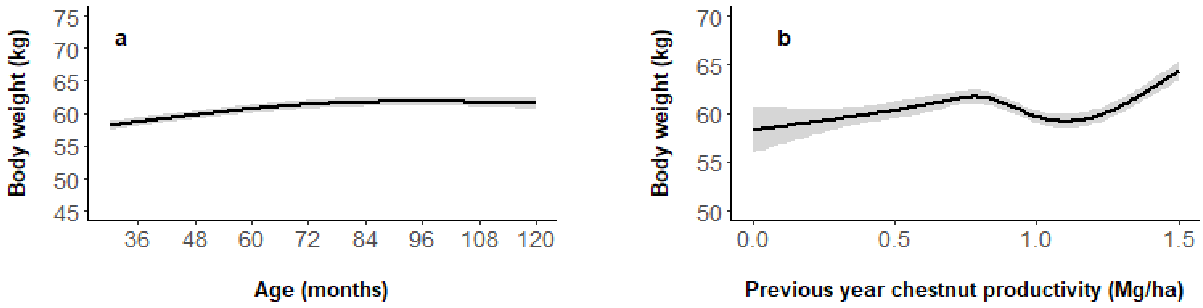

Fig. S2. Effect of other predictor variables (a: individual age, b: previous year chestnut productivity) included in the best generalised additive mixed model explaining adult female body weight variation throughout the hunting season (see the text for more details). Grey-shaded areas represent the estimated standard errors. The predictions are given according to the mean of all other covariates in the model.

Capital-income breeding in wild boar: a comparison between two sexes

R. Brogi, R. Chirichella, F. Brivio, E. Merli, E. Bottero, and M. Apollonio

SUPPLEMENTARY INFORMATION

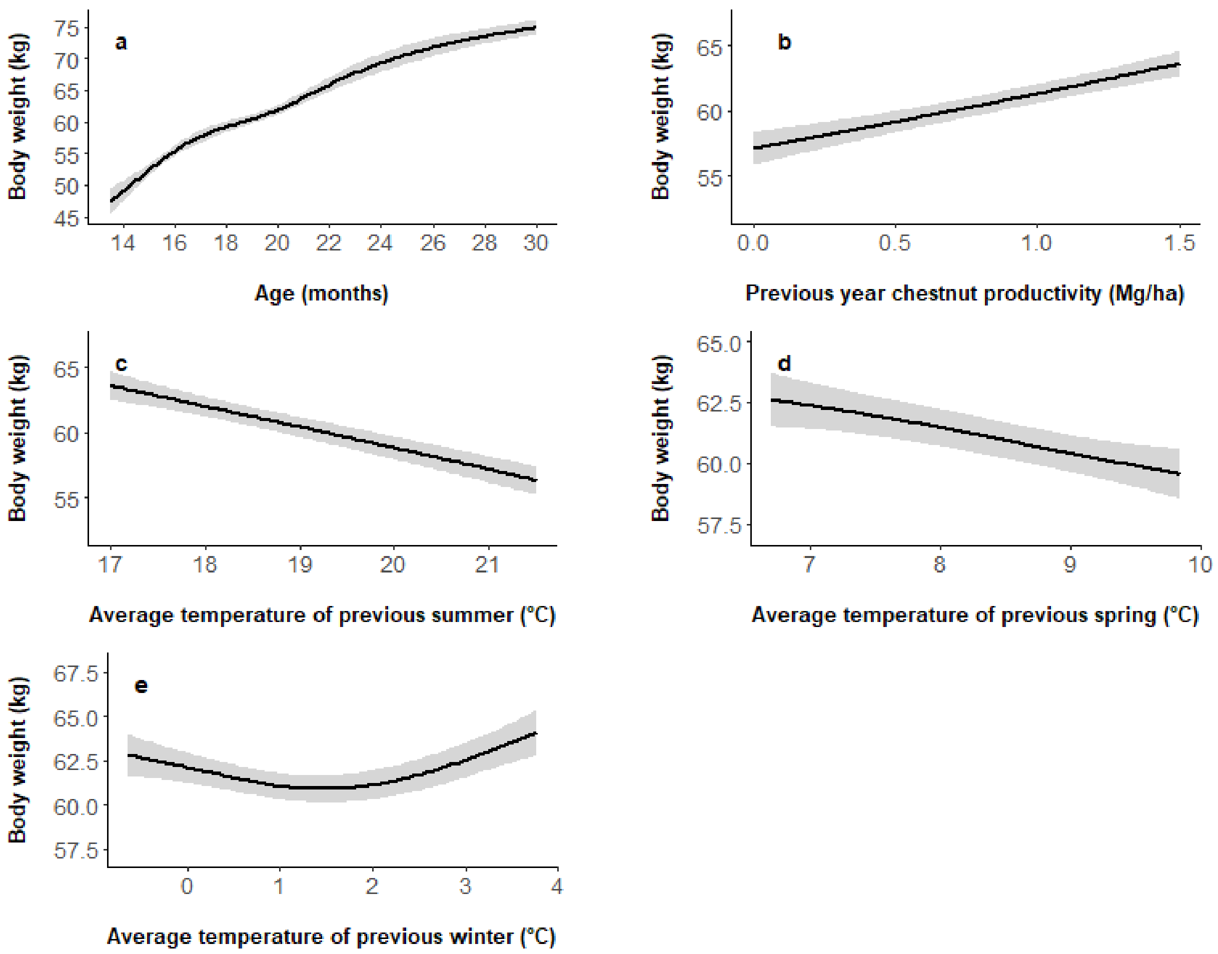

Fig. S3. Effect of other predictor variables (a: individual age, b: previous year chestnut productivity, c: average temperature of previous summer, d: average temperature of previous spring, e: average temperature of previous winter) included in the best generalised additive mixed model explaining subadult male body weight variation throughout the hunting season (see the text for more details). Grey-shaded areas represent the estimated standard errors. The predictions are given according to the mean of all other covariates in the model.

Capital-income breeding in wild boar: a comparison between two sexes

R. Brogi, R. Chirichella, F. Brivio, E. Merli, E. Bottero, and M. Apollonio

SUPPLEMENTARY INFORMATION

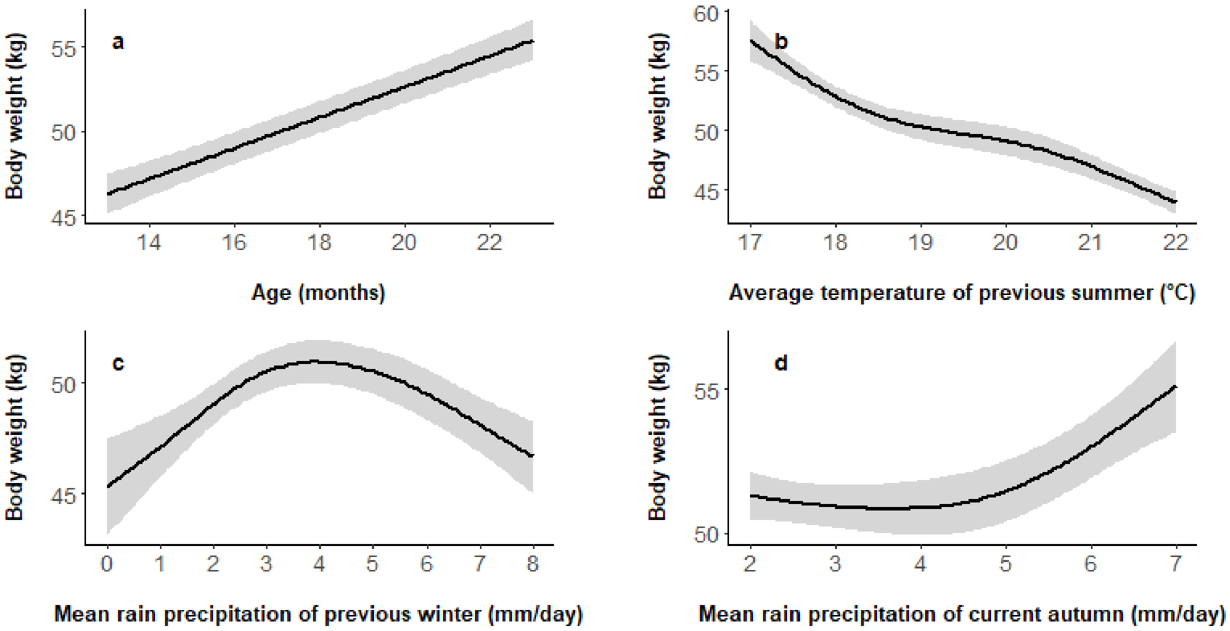

Fig. S4. Effect of other predictor variables (a: individual age, b: average temperature of previous summer, c: mean rain precipitation of previous winter, d: mean rain precipitation of current autumn) included in the best generalised additive mixed model explaining subadult female body weight variation throughout the hunting season (see the text for more details). Grey-shaded areas represent the estimated standard errors. The predictions are given according to the mean of all other covariates in the model.

Capital-income breeding in wild boar: a comparison between two sexes

R. Brogi, R. Chirichella, F. Brivio, E. Merli, E. Bottero, and M. Apollonio

SUPPLEMENTARY INFORMATION

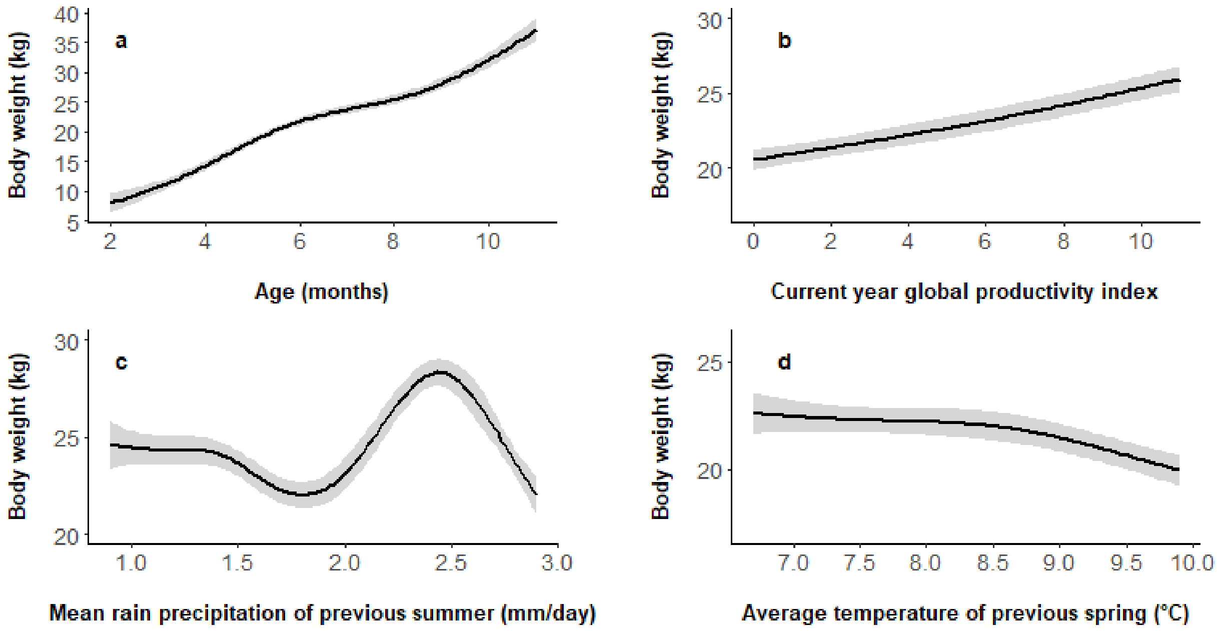

Fig. S5. Effect of other predictor variables (a: individual age, b: current year global productivity index, c: mean rain precipitation of previous summer, d: average temperature of previous spring) included in the best generalised additive mixed model explaining male piglet body weight variation throughout the hunting season (see the text for more details). Grey-shaded areas represent the estimated standard errors. The predictions are given according to the mean of all other covariates in the model.

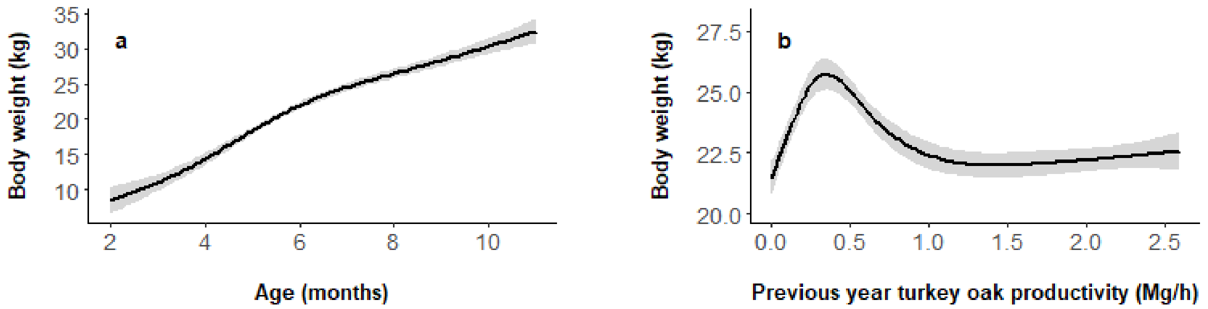

Fig. S6. Effect of other predictor variables (a: individual age, b: previous year Turkey oak productivity) included in the best generalised additive mixed model explaining female piglet body weight variation throughout the hunting season (see the text for more details). Grey-shaded areas represent the estimated standard errors. The predictions are given according to the mean of all other covariates in the model.

Capital-income breeding in wild boar: a comparison between two sexes

R. Brogi, R. Chirichella, F. Brivio, E. Merli, E. Bottero, and M. Apollonio

SUPPLEMENTARY INFORMATION

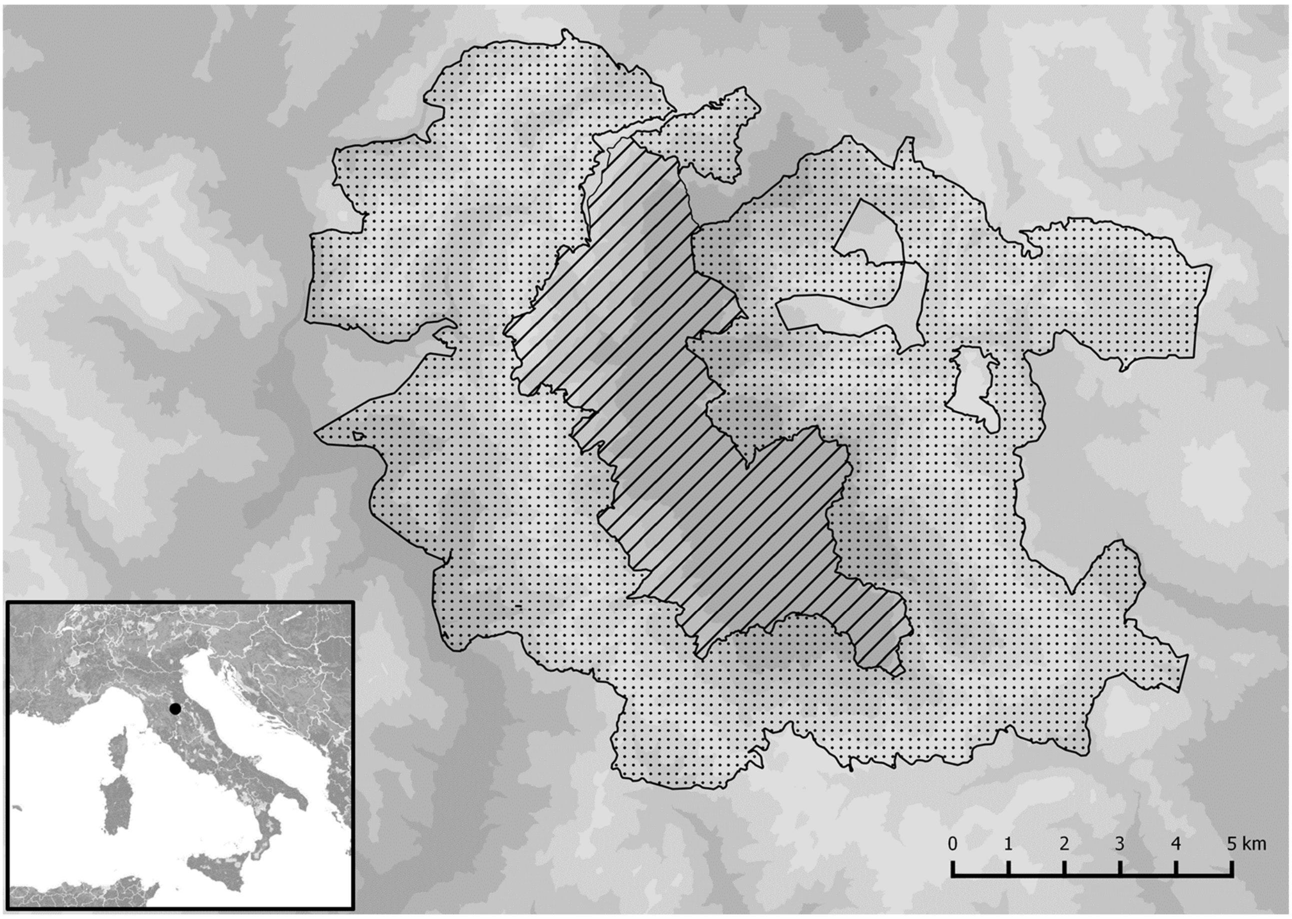

Fig. S7. Study area map showing the hunting area (dotted) enclosing the protected area (Oasi Alpe di Catenaia, striped), with the greyscale representing altitude (dark: higher; light: lower). Bottom-left inset: position of the study area (black dot) in the Italian peninsula.

Capital-income breeding in wild boar: a comparison between two sexes

R. Brogi, R. Chirichella, F. Brivio, E. Merli, E. Bottero, and M. Apollonio

SUPPLEMENTARY INFORMATION

Tab. S1. Gompertz growth models estimated parameters for males and females.

	Parameter	Estimate	p-value	Significance code
Male	*a*	84.69	$<2*10^{-16}$	***
	b	2.55	$<2*10^{-16}$	***
	c	0.90	$<2*10^{-16}$	***
Female	*a*	60.51	$<2*10^{-16}$	***
	b	2.43	$<2*10^{-16}$	***
	c	0.86	$<2*10^{-16}$	***

a = asymptotic body weight (kg); *b* = displacement on the x-axis; *c* = growth rate; p-value is referred to the statistical significance of the estimated parameter; Significance code = *** p-value < 0.001; ** p-value < 0.01; * p-value < 0.05.

Tab. S2. Sample size subdivided by sex and age classes (see the text for more details about age class subdivision).

	Age class	Sample size	Age threshold (months)	Weight at threshold (kg)
Male	Adults	752	36	80
	Subadults	1629	12	42
	Piglets	2017		
	Total males	4398		
Female	Adults	1376	24	56
	Subadults	1318	12	40
	Piglets	1671		
	Total females	4365		
	**Total**	**8763**		

Capital-income breeding in wild boar: a comparison between two sexes

R. Brogi, R. Chirichella, F. Brivio, E. Merli, E. Bottero, and M. Apollonio

SUPPLEMENTARY INFORMATION

Tab. S3. Summary of explanatory variables used in random forest, full model, and best model, their relative importance, p-value, and statistical significance in the best model on body weight seasonal variability of adult males (a), adult females (b), subadult males (c), subadult females (d), male piglets (e), and female piglets (f).

a) **Adult males**	Random forest	Full model	Best model	Importance	p-value	Significance code
Age	x	x	x	6.42	$4.23*10^{-6}$	***
hunting day	x	x	x	7.23	$9.46*10^{-7}$	***
turkey oak productivity (curr.)	x					
beech productivity (curr.)	x	x				
chestnut productivity (curr.)	x	x				
global productivity index (curr.)	x					
turkey oak productivity (prev.)	x					
beech productivity (prev.)	x					
chestnut productivity (prev.)	x	x				
global productivity index (prev.)	x	x				
winter average temperature	x					
spring average temperature	x	x	x	3.45	$4.71*10^{-7}$	***
summer average temperature	x					
autumn average temperature	x					
winter daily rain precipitation	x	x	x	0.92	$2.36*10^{-2}$	*
spring daily rain precipitation	x					
summer daily rain precipitation	x	x				
autumn daily rain precipitation	x					

b) **Adult females**	Random forest	Full model	Best model	Importance	p-value	Significance code
Age	x	x	x	13.95	$<2*10^{-16}$	***
hunting day	x	x	x	47.90	$<2*10^{-16}$	***
turkey oak productivity (curr.)	x					
beech productivity (curr.)	x	x				
chestnut productivity (curr.)	x					
global productivity index (curr.)	x					
turkey oak productivity (prev.)	x	x				
beech productivity (prev.)	x	x				
chestnut productivity (prev.)	x	x	x	6.39	$1.81*10^{-5}$	***
global productivity index (prev.)	x					
winter average temperature	x					
spring average temperature	x	x				

Capital-income breeding in wild boar: a comparison between two sexes

R. Brogi, R. Chirichella, F. Brivio, E. Merli, E. Bottero, and M. Apollonio

SUPPLEMENTARY INFORMATION

summer average temperature	x	x				
autumn average temperature	x	x				
winter daily rain precipitation	x	x				
spring daily rain precipitation	x					
summer daily rain precipitation	x	x				
autumn daily rain precipitation	x					

**c) Subadult males**	Random forest	Full model	Best model	Importance	p-value	Significance code
Age	x	x	x	99.48	$<2*10^{-16}$	***
hunting day	x	x	x	4.02	$4.55*10^{-3}$	**
turkey oak productivity (curr.)	x					
beech productivity (curr.)	x					
chestnut productivity (curr.)	x					
global productivity index (curr.)	x	x				
turkey oak productivity (prev.)	x					
beech productivity (prev.)	x					
chestnut productivity (prev.)	x	x	x	2.20	$1.96*10^{-6}$	***
global productivity index (prev.)	x	x				
winter average temperature	x	x	x	0.86	$1.12*10^{-2}$	*
spring average temperature	x	x	x	0.62	$3.82*10^{-3}$	**
summer average temperature	x	x	x	3.33	$<2*10^{-16}$	***
autumn average temperature	x					
winter daily rain precipitation	x	x				
spring daily rain precipitation	x					
summer daily rain precipitation	x	x				
autumn daily rain precipitation	x					

**d) Subadult females**	Random forest	Full model	Best model	Importance	p-value	Significance code
Age	x	x	x	17.35	$<2*10^{-16}$	***
hunting day	x	x	x	19.51	$<2*10^{-16}$	***
turkey oak productivity (curr.)	x					
beech productivity (curr.)	x					
chestnut productivity (curr.)	x					
global productivity index (curr.)	x					
turkey oak productivity (prev.)	x	x				
beech productivity (prev.)	x					
chestnut productivity (prev.)	x					
global productivity index (prev.)	x					

Capital-income breeding in wild boar: a comparison between two sexes

R. Brogi, R. Chirichella, F. Brivio, E. Merli, E. Bottero, and M. Apollonio

SUPPLEMENTARY INFORMATION

winter average temperature	x	x				
spring average temperature	x	x				
summer average temperature	x	x	x	9.64	$<2*10^{-16}$	***
autumn average temperature	x					
winter daily rain precipitation	x	x	x	1.25	$2.07*10^{-3}$	**
spring daily rain precipitation	x					
summer daily rain precipitation	x	x				
autumn daily rain precipitation	x	x	x	1.22	$1.14*10^{-3}$	**

**e) Male piglets**	Random forest	Full model	Best model	Importance	p-value	Significance code
Age	x	x	x	91.44	$<2*10^{-16}$	***
hunting day	x	x	x	16.35	$<2*10^{-16}$	***
turkey oak productivity (curr.)	x	x				
beech productivity (curr.)	x					
chestnut productivity (curr.)	x					
global productivity index (curr.)	x	x	x	5.34	$<2*10^{-16}$	***
turkey oak productivity (prev.)	x	x				
beech productivity (prev.)	x					
chestnut productivity (prev.)	x	x				
global productivity index (prev.)	x	x				
winter average temperature	x					
spring average temperature	x	x	x	1.42	$2.01*10^{-4}$	***
summer average temperature	x					
autumn average temperature	x	x				
winter daily rain precipitation	x	x				
spring daily rain precipitation	x					
summer daily rain precipitation	x	x	x	10.17	$<2*10^{-16}$	***
autumn daily rain precipitation	x					

**f) Female piglets**	Random forest	Full model	Best model	Importance	p-value	Significance code
Age	x	x	x	68.12	$<2*10^{-16}$	***
hunting day	x	x	x	35.16	$<2*10^{-16}$	***
turkey oak productivity (curr.)	x	x				
beech productivity (curr.)	x					
chestnut productivity (curr.)	x					
global productivity index (curr.)	x	x				
turkey oak productivity (prev.)	x	x	x	10.41	$<2*10^{-16}$	***
beech productivity (prev.)	x					

Capital-income breeding in wild boar: a comparison between two sexes

R. Brogi, R. Chirichella, F. Brivio, E. Merli, E. Bottero, and M. Apollonio

SUPPLEMENTARY INFORMATION

chestnut productivity (prev.)	x	x				
global productivity index (prev.)	x	x				
winter average temperature	x					
spring average temperature	x	x				
summer average temperature	x					
autumn average temperature	x					
winter daily rain precipitation	x	x				
spring daily rain precipitation	x					
summer daily rain precipitation	x	x				
autumn daily rain precipitation	x	x				

x = the explanatory variable was included; empty cell = the explanatory variable was not included; age = individual age expressed in months; hunting day = culling date expressed as days from 1 September; productivity of species *x* (curr.) = mast productivity of the tree species *x* during the current year expressed as Mg/ha; global productivity index (curr.) = index summarising all tree species productivity during the current year (see the text for more details); productivity of species *x* (prev.) = mast productivity of the tree species *x* during the previous year expressed as Mg/ha; global productivity index (prev.) = index summarising all tree species productivity during the previous year; season *y* average temperature = average environmental temperature recorded during the season *y* (see the text for more details); season *y* daily rain precipitation = average daily rain precipitation recorded during the season *y* (see the text for more details); Random forest = explanatory variables selection process; Full model = GAM including all the explanatory variables selected; Best model = best alternative model selected following the minimum AIC criterion; Importance = importance of the predictor variable in explaining individual body weight variability, calculated as the F statistic; p-value is referred to the statistical significance of the predictor variable in explaining body weight variability; Significance code = *** p-value < 0.001; ** p-value < 0.01; * p-value < 0.05.
